# Msi1 promotes tumor growth and cell proliferation by targeting cell cycle checkpoint proteins p21, p27 and p53 in cervical carcinomas

**DOI:** 10.18632/oncotarget.2539

**Published:** 2014-10-24

**Authors:** Xian Liu, Wen-Ting Yang, Peng-Sheng Zheng

**Affiliations:** ^1^ Department of Reproductive Medicine, The First Affiliated Hospital of the Medical College, Xi'an Jiaotong University, Xi'an, The People's Republic of China; ^2^ Section of Cancer Stem Cell Research, Key Laboratory of Environment and Genes Related to Diseases, Ministry of Education of the People's Republic of China, Xi'an, The People's Republic of China

**Keywords:** Msi1, cervical cancer, cell cycle, oncogene

## Abstract

Musashi RNA-binding protein1 (Msi1), a member of the RNA-binding protein family, has been reported to be a diagnostic marker and potential therapeutic target in some cancers, its function in cervical cancer remains unknown. In this study, we found Msi1 was highly expressed in cervical cancer tissues, and over-expressing Msi1 in cervical cancer cells enhanced tumor formation and cell proliferation and accelerated cells into the S phase. Whereas, down-regulating Msi1 by shRNA in cervical cancer cells inhibited tumor formation and cell proliferation and slowed cell into the S phase, suggesting that Msi1 might act as cell cycle regulator. Immunohistochemistry assay showed the negative correlation between Msi1 and p21, p27 and p53, suggesting that Msi1 might regulate these cycle regulators in cervical cancer. Moreover, the expression of the p21, p27 and p53 proteins were down-regulated in Msi1 overexpressing cervical cancer cells and up-regulated in shMsi1 cervical cancer cells. Luciferase assays and RNA-protein binding assays confirmed that Msi1 could bind to the mRNA 3′UTRs of p21, p27 and p53 and suppress the translation of these proteins. Our findings provide new evidence that Msi1 might promote cell proliferation by accelerating the cell cycle by directly targeting p21, p27 and p53.

## INTRODUCTION

Although Papanicolaou testing or other types of screening tests have been promoted, cervical cancer remains the third most commonly diagnosed cancer and the fourth leading cause of cancer death in females worldwide; more than 85% of these cases and deaths occur in developing countries [1]. The increasing morbidity of cervical cancer among young women and the refractoriness of terminal cervical cancer appear to be new problems. Although 99.7% of cervical cancer cases were found to be associated with HPV (human papillomavirus) infection [2], the interactions of multiple factors *in vivo* and *in vitro* that activate oncogenes and inactivate cancer suppressor genes could not be ignored in the long process of cervical cancer development. SOX2 has been reported to be a potential nuclear marker of stem cells in cervical cancer [3]. High ALDH1 activity might be a cytoplasmic marker for cervical cancer stem cells (CCSCs) [4]. ITGA6 (CD49f) might be a possible surface marker of cervical cancer stem cells [5]. Several stem cell related transcription factors, such as OCT4, SOX2, NANOG, KLF4 and UTF1[6], are involved in cervical carcinogenesis [7–10].

Msi1 is a RNA-binding protein of the Musashi family; the preferential binding to the motif was determined to be (G/A)UnAGU where n=1–3[11]. Msi1 has been found to be highly enriched in the nervous system[12] and closely related to the stemness of neural cells. High expression levels of Msi1 were shown to be correlated with the grade of the malignancy in glioma, and primary central nervous system (CNS) tumors might share gene expression patterns with primitive, undifferentiated CNS cells[13, 14]. Additionally, Msi1 was found to drive progenitor cell expansion along the luminal and myoepithelial lineages in mammary glands and to regulate the proliferation and apoptosis of mesenchymal stem cells [15–17]. In recent years, the overexpression of Msi1 has been observed in many malignant tumors that appeared to be associated with a poor prognosis, such as medulloblastoma[18, 19], colon cancer[20–22], gastric cancer[23, 24], lung cancer[25], breast cancer[26] and endometrial cancer[27–29]. Abreu used in-depth literature mining with Pathway Studio to reveal that Msi1-associated genes were mainly involved in cell proliferation (39%), cell differentiation (36%), cell cycle (36%), and apoptosis (33%) [30]. The role of Msi1 in cervical cancer is unknown, and the molecular mechanisms of cervical carcinoma are not fully understood. This study aimed to fully explore the function and mechanism of Msi1 in cervical carcinogenesis.

## RESULTS

### The expression of msi1 in human normal cervix samples and various cervical cancer lesions

**Figure 1:**
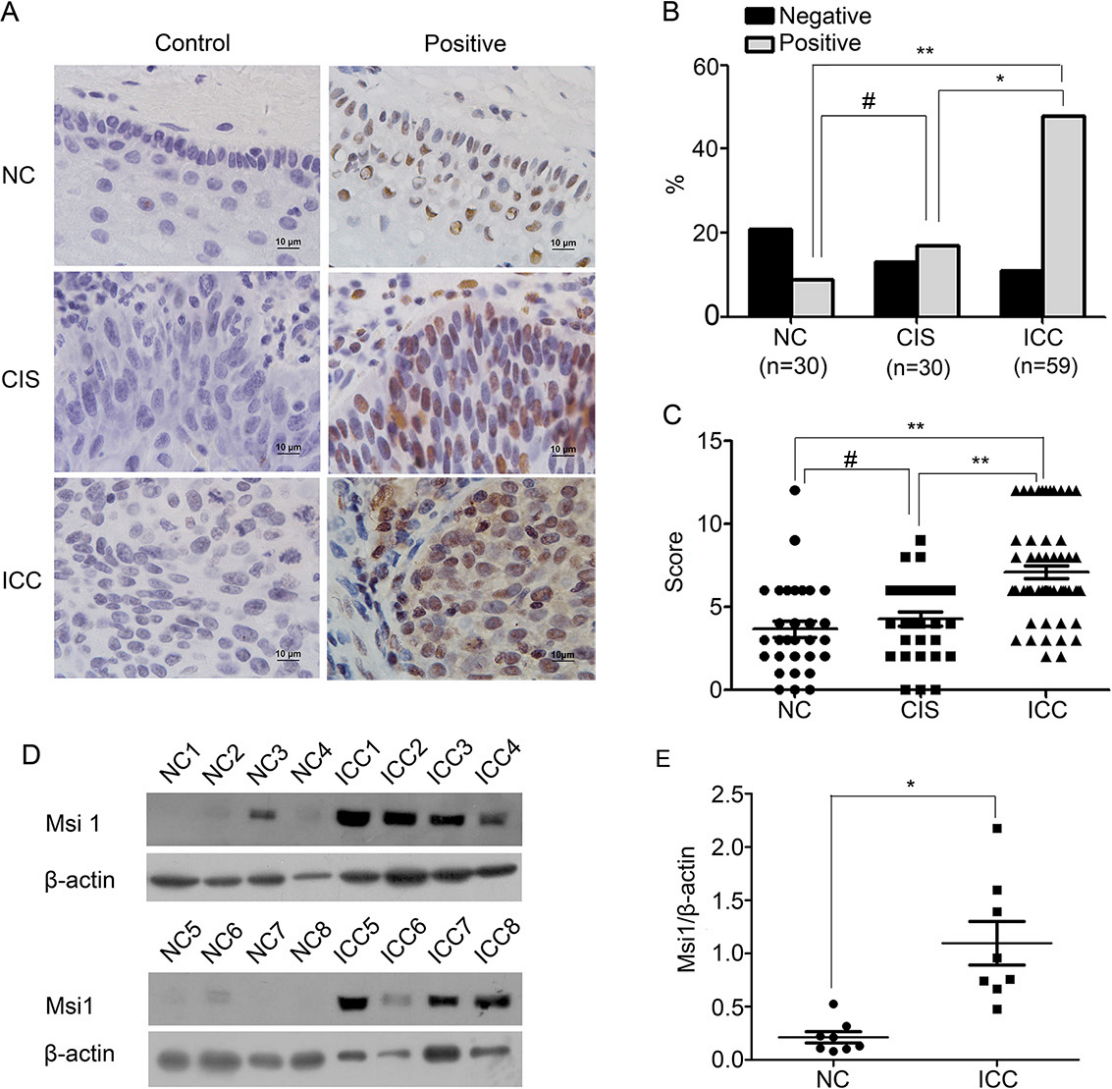
Msi1 expression is shown in normal cervix samples and in various cervical lesions **(A)** Immunohistochemistry (IHC) for Msi1 expression is shown in a normal cervix sample, cancer in situ, and cervical carcinoma; original magnification, ×1000. **(B)** Msi1 staining is classified into 2 categories (negative and positive), and the percentage of each group is shown for 30 normal cervix specimens, 30 cervical cancer in situ specimens, and 59 invasive cervical cancer specimens. **(C)** A comparison of the IHC scores of Msi1 staining in normal cervix, cervical cancer in situ, and invasive cervical cancer is shown (points represent the IHC score per specimen). **(D)** Representative Western blots of Msi1 proteins in normal cervix samples (NC) and invasive cervical cancer samples (ICC) are shown. **(E)** The protein abundance of the Msi1/β-actin ratio in each normal cervix tissue sample (n=8) and invasive cervical cancer tissue sample (n=8) is shown. The data shown are the Msi1 relative expression levels of each specimen and the means± standard error of the NC and ICC groups (points represent the Msi1 relative expression). *P < 0.05; **P < 0.01; #P>0.05.

Although Msi1 expression has been discovered in various carcinomas[13, 18, 20, 23], its role in cervical cancer is not well defined. In the present study, the expression of Msi1 was detected by immunohistochemistry in normal cervix (NC), cervical carcinoma in situ (CIS) and in invasive cervical carcinoma (ICC) samples (Fig. 1A-1C). Msi1 positive staining localized in nucleus and/or cytoplasm (Fig. 1A) was found in 30% (9 of 30) of the NC samples, in 43.3% (13 of 30) of the CIS samples and in 81.4% (48 of 59) of the ICC samples (Fig. 1B, NC vs CIS, P>0.05; NC vs ICC, P<0.001; CIS vs ICC, P<0.05). The average scores of IHC for Msi1 were 3.67±2.72 in NC, 4.27±2.39 in CIS, 7.10±2.90 in ICC (Fig. 1C, NC vs CIS, P>0.05; NC vs ICC, P<0.001; CIS vs ICC, P<0.001). These data suggested that Msi1 is involved in the progression, although not the development, of cervical carcinomas. Furthermore, Western blot analyses were performed to examine Msi1 expression in 8 randomly selected NC samples and ICC fresh specimens (Fig. 1D). The relative expression level of Msi1 in these cervical cancer samples was higher than that in the normal cervical tissues (Fig. 1E, P<0.05). All of these results indicated that Msi1 was up-regulated in cervical carcinoma.

### Msi1 enhances the tumor formation of cervical cancer cells *in vivo*

**Figure 2:**
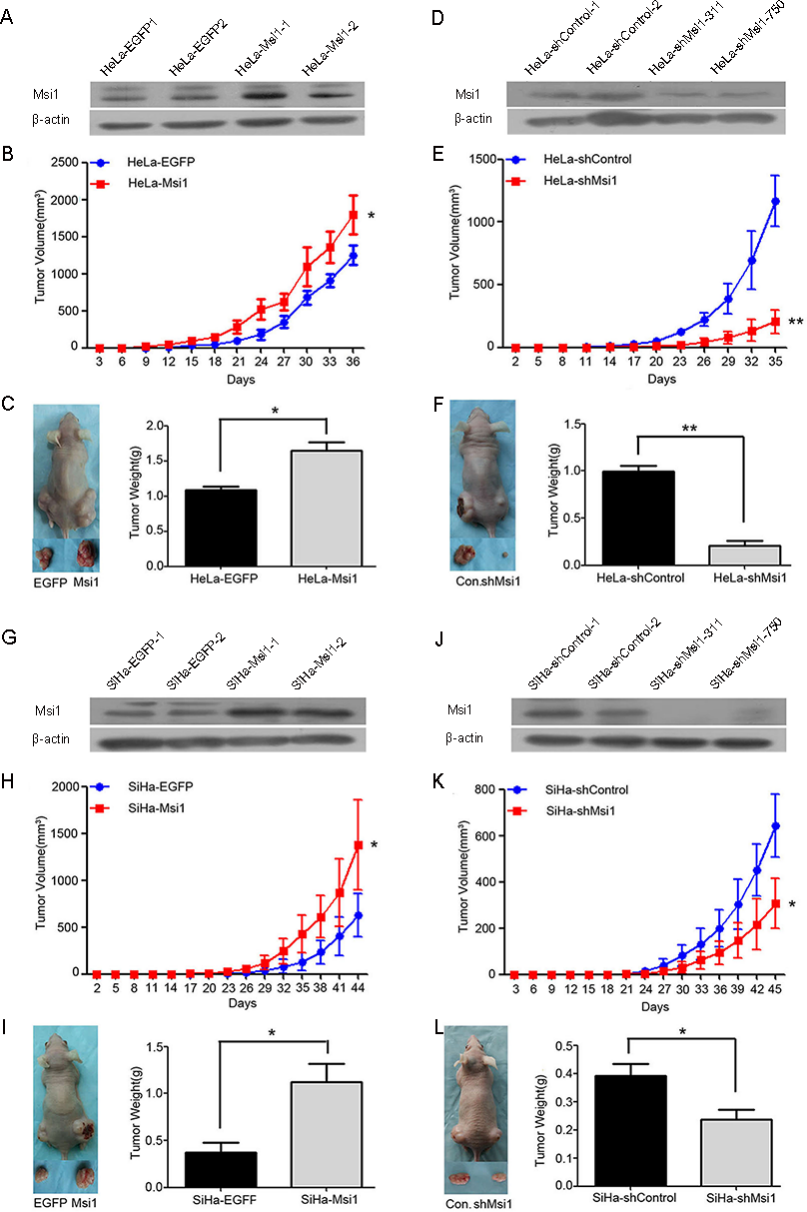
Msi1 enhances tumor formation by cervical cancer cells *in vivo* Western blot analysis of Msi1 protein in HeLa-EGFP and HeLa-Msi1 cells **(A)** HeLa-shControl and HeLa-shMsi1 cells **(D)** SiHa-EGFP and SiHa-Msi1 cells **(G)** and SiHa-shControl and SiHa-shMsi1 cells **(J)** β-actin served as the loading control. Tumor formation assays were performed with 6 mice per group. The tumor growth curves were calculated by monitoring that was performed every 3 days post-transplant. At 36 days post-transplant for HeLa-Msi1 cells **(B)** 35 days post-transplant for HeLa-shMsi1 cells **(E)** 44 days post-transplant for SiHa-Msi1 cells **(H)** and 45 days post-transplant for SiHa-shMsi1 cells **(K)** the xenograft tumors of mice in each group were dissociated and weighed **(C, F, I and L)**. The data are shown as the mean± S.E.M. *P < 0.05; **P < 0.01.

Msi1 was found by immunocytochemistry and Western blot analysis to be expressed in all four cervical cancer cell lines (SiHa, HeLa, C33A and Caski) (Supplement Fig. A-C). Msi1 expression in HeLa and SiHa cells was up-regulated by stably transfecting a constructed Msi1-expressing plasmid (Fig. 2A, 2D) and down-regulated by stably transfecting a shRNA targeting Msi1 plasmid (Fig. 2G, 2J). A total of 10^6^ of the control and Msi1-modified HeLa and SiHa cells were subcutaneously inoculated into each posterior flank of the same female nude mouse(six mice per group) at the same time for the tumor formation assay. The HeLa-Msi1 cells xenographed a tumor much faster (a palpable tumor took 12 days for HaLa-Msi1 cells and 18 days for HeLa-EGFP cells), and the tumor was larger (Fig. 2B; P<0.05) and heavier (Fig. 2C; P<0.05) than with the HeLa-EGFP control cells. Similarly, the SiHa-Msi1 cells formed a tumor much faster (a palpable tumor took 23 days for SiHa-Msi1 cells and 29 days for SiHa-EGFP cells), and the tumor was larger (Fig. 2H; P<0.05) and heavier (Fig. 2I; P<0.05) than with the SiHa-EGFP control cells. Furthermore, the HeLa-shMsi1 cells produced a tumor much slower (a palpable tumor took 23 days for HaLa-shMsi1 cells and 17 days for HeLa-shControl cells), and the tumor was smaller (Fig. 2E; P<0.05) and lighter (Fig. 2F; P<0.05) than with the HeLa-shControl cells. Similarly, SiHa-shMsi1 cells developed a tumor slower (a palpable tumor took 30 days for SiHa-shMsi1 cells and 24 days for SiHa-shControl cells), and the tumor was smaller (Fig. 2K; P<0.05) and lighter (Fig. 2 L; P<0.05) than with the SiHa-shControl cells did. All of these results indicated that the Msi1 protein enhanced the tumor formation of cervical cancer cells *in vivo*.

### Msi1 promoted the proliferation of cervical cancer cells *in vitro* as well as *in vivo*

**Figure 3:**
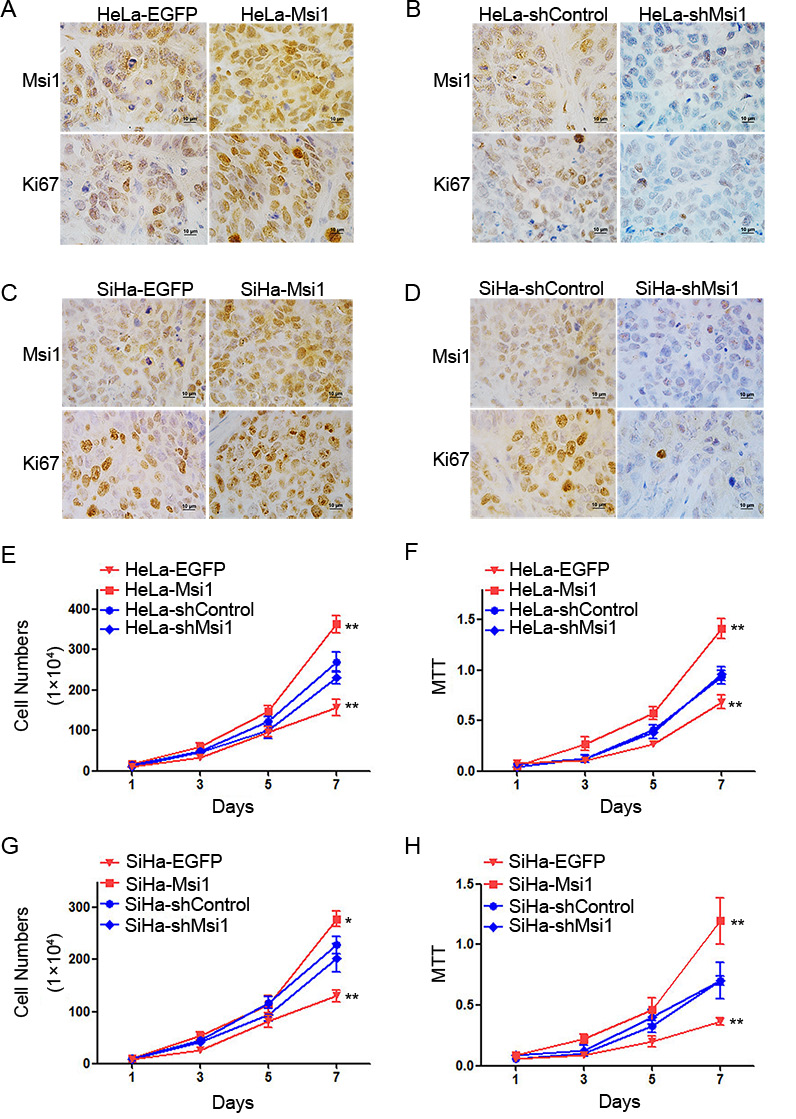
Msi1 promotes the proliferation of cervical cancer cells *in vitro* and *in vivo* Immunochemistry for Msi1 and Ki-67 is shown in tumor xenografts of HeLa-EGFP and HeLa-Msi1 cells **(A)** HeLa-shControl and HeLa-shMsi1 cells **(B)** SiHa-EGFP and SiHa-Msi1 cells **(C)** and SiHa-shControl and SiHa-shMsi1 cells **(D)**. Cell proliferation in vivo was determined by counting the cells for Msi1-modified HeLa cells **(E)** and Msi1-modified SiHa cells **(G)**. A cell viability assay (3-[4,5-dimethylthiazole-yl]-2,5-diphenyl tetrazolium bromide) was performed for Msi1-modified HeLa cells **(F)** and Msi1-modified SiHa cells **(H)**. The data are presented as the mean± S.E.M. *P < 0.05; **P < 0.01.

To further explore why Msi1 enhanced the tumor formation of cervical cancer cells *in vivo*, the expression of Ki67 was evaluated by immunohistochemistry in the tumor tissues xenographed by the control and Msi1-modified HeLa and SiHa cells. Ki67 staining was found to express much stronger in the tumor tissues formed by the Msi1-overexpressing HeLa (Fig. 3A) and SiHa (Fig. 3C) cells than in the tumors formed by the control cells. Furthermore, the Ki67 protein was much weaker in the tumor tissues xenographed by the shMsi1-transfected HeLa (Fig. 3B) and SiHa (Fig. 3D) cells than in the tumors formed by the shControl cells. These results suggested that Msi1 enhanced tumor formation by promoting the proliferation of cervical cancer cells *in vivo*.

To confirm the effect of Msi1 on the proliferation of cervical cancer cells *in vitro*, a cell growth curve assay and MTT assay were used to evaluate the cell growth and viability of the control and Msi1-modified HeLa and SiHa cells, respectively. A cell growth curve assay showed that both HeLa- and SiHa-Msi1 cells had much stronger effects on cell growth than did their control EGFP cells (Fig. 3E and 3G,P<0.05). Similarly, the shMsi1-transfected HeLa and SiHa cells were found to have much weaker effects on cell growth than did their control groups (Fig. 3E and 3G, P<0.05). Furthermore, the MTT assays showed that both HeLa-, and SiHa-Msi1 cells displayed much higher cell viability than their control EGFP cells (Fig. 3F and 3H, P<0.05). Similarly, the shMsi1-transfected HeLa and SiHa cells were found to have much lower cell viability than their control groups (Fig. 3F and 3H, P<0.05). All of these data indicated that Msi1 expression could promote the proliferation of cervical cancer cells *in vitro*.

### Msi1 accelerated the transition of cervical cancer cells from G0/G1 into S phase

**Figure 4:**
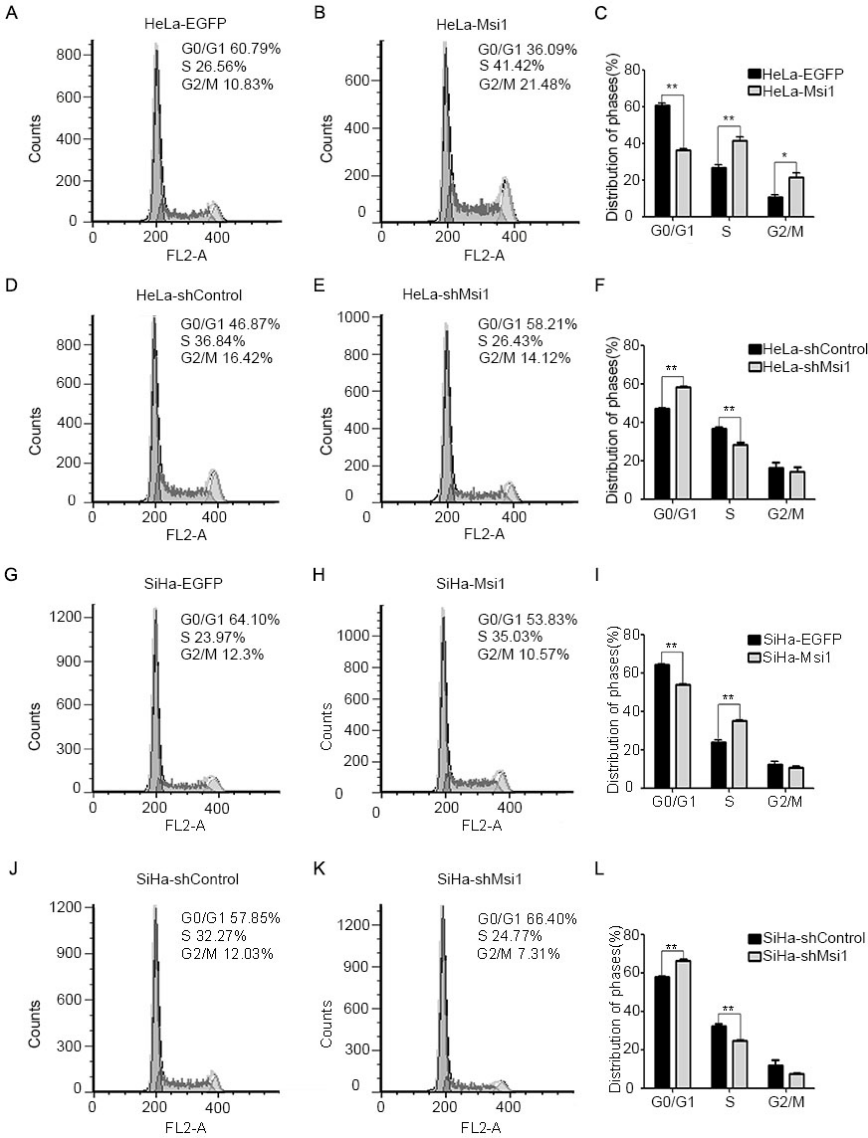
Msi1 accelerates the transition of the cell cycle from the G0/G1 phase into the S phase in cervical cancer cells The cell cycles of HeLa-EGFP **(A)** and HeLa-Msi1 **(B)** were monitored with fluorescence-activated cell sorting (FACS) analysis, and a quantitative analysis of the cell cycle distribution is shown, respectively **(C)**. The cell cycles of HeLa-shControl **(D)** and HeLa-shMsi1 **(E)** were monitored with FACS analysis, and a quantitative analysis of the cell cycle distribution is shown, respectively **(F)**. The cell cycles of SiHa-EGFP **(G)** and SiHa-Msi1 **(H)** were monitored with FACS analysis, and a quantitative analysis for the cell cycle distribution is shown, respectively **(I)**. SiHa-shControl **(J)** and SiHa-shMsi1 **(K)** cells were monitored with FACS analysis, and a quantitative analysis of the cell cycle distribution is shown, respectively (L). The data were analyzed and are presented as the mean±S.E.M. *P < 0.05, **P < 0.01.

To investigate how the Msi1 protein affects cell proliferation of cervical cancer cells, fluorescence-activated cells sorting (FACS) was used to analyze the cell cycle of the control and Msi1-modified HeLa and SiHa cells. The representative FACS figures for the control cells were shown in Fig. 4A, D, G, J, and the figures for the Msi1-modified cells were shown in Fig. 4B, E, H, K); the FACS data were summarized in Fig. 4C, F, I, L, respectively. The Msi1-overexpressing HeLa cells had 36.09% of G0/G1 phase cells, which was much lower than the number of HeLa control EGFP cells in the G0/G1 phase (60.79%) (Fig.4C; P<0.01). However, 41.42% of the HeLa-Msi1 cells were in the S phase, which was much higher than number of HeLa-EGFP cells in the S phase (26.56%) (Fig. 4C; P<0.01). Furthermore, the Msi1-overexpressing SiHa cells had 53.83% of G0/G1 phase cells, which was much lower than the SiHa control EGFP cells (64.10%)(Fig. 4I; P<0.01); 35.03% of the SiHa-Msi1 cells were in the S phase, which was much higher than the SiHa-EGFP cells (23.97%)(Fig. 4I; P<0.01). These results suggested that Msi1 expression promoted cervical cancer cells from the G0-G1 phase into the S phase. Fig. 4F showed that 58.21% of the HeLa-shMsi1 cells were in the G0/G1 phase, and 28.43% were in the S phase cells, whereas of the HeLa-shControl cells, 46.87% were in the G0/G1 phase and 36.84% were in the S phase (P<0.01). Fig. 4L showed that 66.40% of the SiHa-shMsi1 cells were in the G0/G1 phase, and 24.77% were in the S phase cells, whereas 57.85% of the SiHa-shControl cells were in the G0/G1 phase, and 32.27% were in the S phase (P<0.01). These results suggested that a lack of Msi1 might inhibit cervical cancer cells from transitioning from the G0-G1 phase into the S phase. All of these results indicated that Msi1 was a positive pivotal regulator of the cell cycle in cervical cancer cells.

### Msi1 limited the expression of the cell cycle regulators p21, p27 and p53 in cervical cancer cells

**Figure 5:**
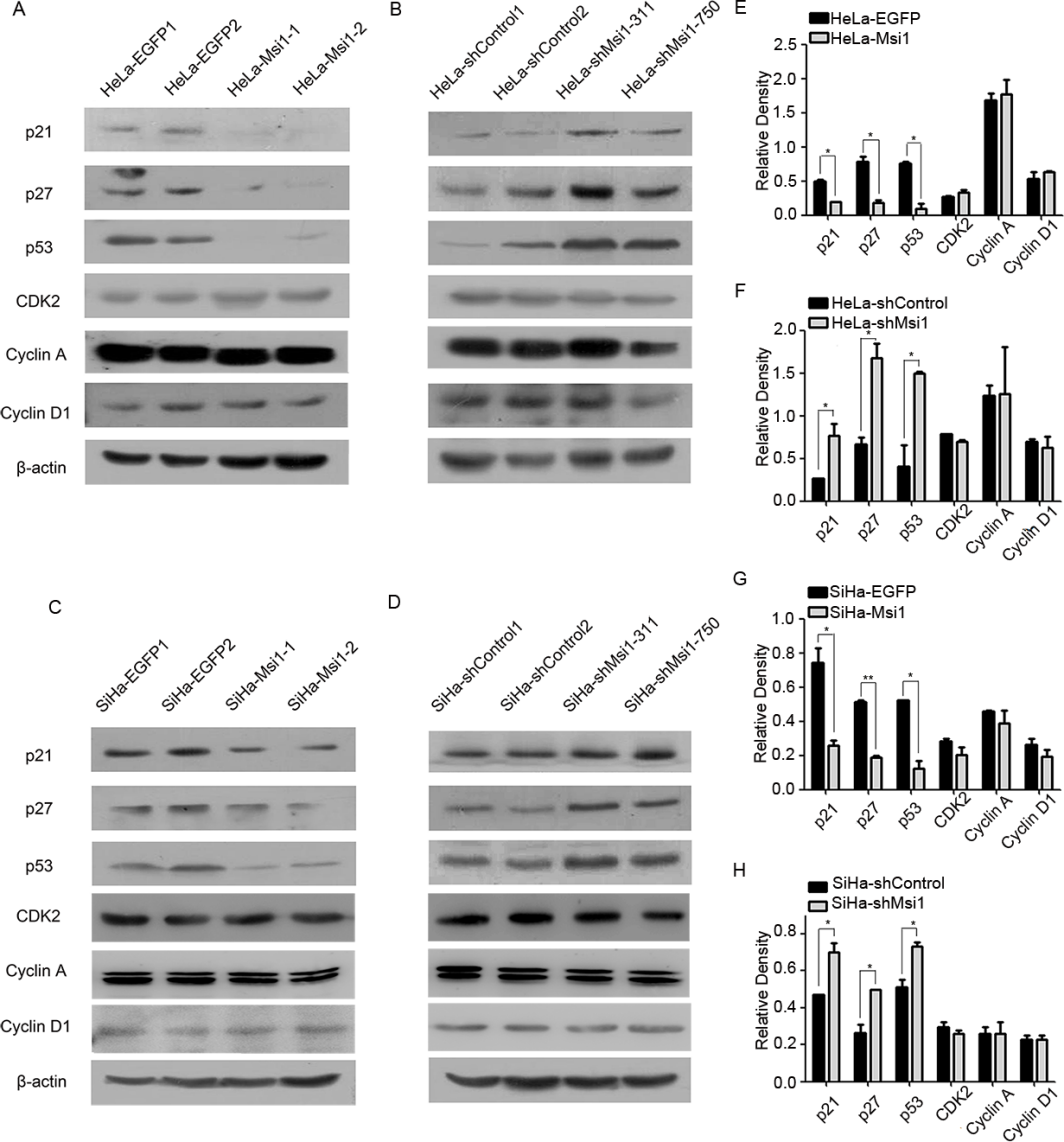
Msi1 represses the expression of the cell cycle regulators p21, p27 and 53 in cervical cancer cells Western blot analysis showing the changes of the endogenous protein levels of p21, p27, p53, CDK2, cyclin A and cyclin D1 in HeLa-EGFP and HeLa-Msi1 cells **(A)** with the quantitative analysis **(E)** HeLa-shControl and HeLa-shMsi1 cells **(B)** with the quantitative analysis **(F)** SiHa-EGFP and SiHa-Msi1 cells **(C)** with the quantitative analysis **(G)** and SiHa-shControl and SiHa-shMsi1 cells **(D)** with the quantitative analysis **(H)**. The data are shown as the mean± S.E.M. *P < 0.05, **P < 0.01.

To explore the molecular mechanism involved in the function of Msi1 on the cell cycle of cervical cancer cells, the mRNA levels of several key cell cycle regulators (p21, p27, p53, CDK2, cyclinA and cyclinD1) were examined by Real-Time PCR in Msi1-overexpressing and Msi1-knockdown cervical cancer cells (HeLa-Msi1, SiHa-Msi1, HeLa-shMsi1 and SiHa-shMsi1 cells) as well as in the control cells (HeLa-EGFP, SiHa-EGFP, HeLa-shControl and SiHa-shControl cells). However, there was no significant difference found between these cells (supplement Fig. D and E), suggesting that the function of Msi1 might not affect these cell cycle regulators at the transcriptional level. Furthermore, because Msi1 is a RNA-binding protein and might affect some proteins at the translational level [31–33], a Western blot assay was performed to examine the protein expression of these cell cycle regulators (p21, p27, p53, CDK2, cyclinA and cyclinD1) in these Msi1-modified cells. The representative Western blot results of these cell cycle regulators (p21, p27, p53, CDK2, cyclinA and cyclinD1) between the Msi1 modified cells and their control cells are shown in Fig. 5A-5D, and their quantitative data are summarized in Fig. 5E-5H. There were no significant protein expression differences for Cyclin A, CyclinD1 and CDK2. However, a significant reduction in the protein levels of p21 (P<0.05), p27 (P<0.05) and p53 (P<0.05) were found in the Msi1 overexpressing SiHa and HeLa cells (Fig. 5E and 5G) compared with their control cells, respectively. An elevation at the protein level of p21 (P<0.05), p27 (P<0.05) and p53 (P<0.05) was observed in shMsi1 HeLa and SiHa cells (Fig. 5F and 5H) compared with their control cells, respectively. All of these results further confirmed that Msi1 limited the protein expression of p21, p27 and p53 at the translational level.

### Msi1 binds to the 3′utr of p21, p27 and p53 and represses their translation

**Figure 6:**
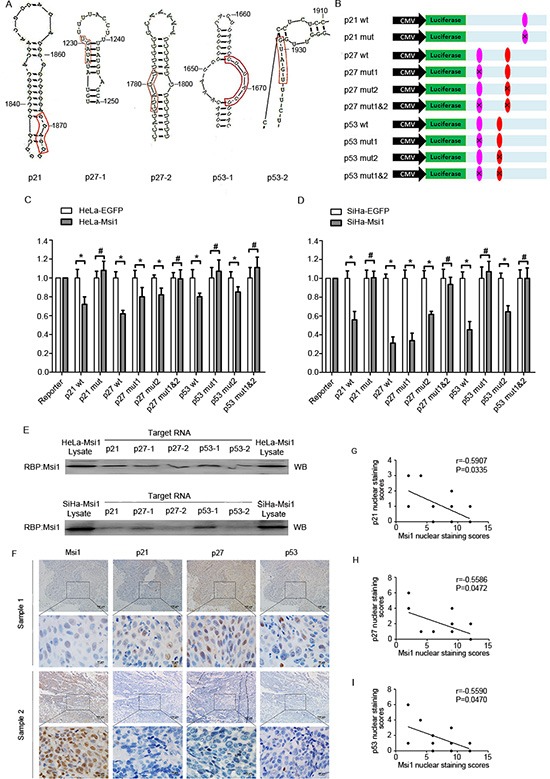
Msi1 Binds to the 3′UTR of p21, p27 and p53 and represses their translation **(A)** Secondary RNA structure surrounding the Msi1 binding sites in p21, p27 and p53 as predicted by the RNAstructure 4.6 software. p21 has only one proven Msi1 binding site, p27 has two predicted Msi1 binding sites (p27–1 and p27–2), and p53 has two predicted Msi1 binding sites (p53–1 and p53–2). **(B)** Schematic representation of the reporter construct containing the firefly luciferase coding sequence fused to the p21 3′UTR/p27 3′UTR/p53 3′UTR. The predicted binding site regions within the wild-type p21 3′UTR/p27 3′UTR/p53 3′UTR are noted in colored ovals, and the residues altered in the p21 3′UTR/p27 3′UTR/p53 3′UTR mutants construct are marked on the ovals. Luciferase reporter assays are shown for p21, p27 and p27 targeted by Msi1 in HeLa-Msi1 **(C)** and SiHa-Msi1 cells **(D)**. The histogram shows the ratio of firefly to Renilla luciferase activity normalized to empty vector-transfected cells (baseline). The data are shown as the mean± S.E.M. *P < 0.05. **(E)** The RNA-Msi1 binding assay shows associations of binding sites of p21, p27–1, p27–2, p53–1 and p53–2 with Msi1 protein in HeLa-Msi1 cells(upper) and SiHa-Msi1 cells(lower). **(F)** The expression of Msi1 and p21, p27 and p53 in 13 cervical cancers specimens. The expression levels of two representative samples are shown. The Msi1, p21, p27 and p53 staining were scored from 1 to 12. The correlation was determined by Pearson's correlation test. **(G)** The correlation between Msi1 and p21 was significant (r=-0.5907, P=0.0335). **(H)** The correlation between Msi1 and p27 was significant (r=-0.5586, P=0.0472). **(I)** The correlation between Msi1 and p53 was significant (r=-0.5590, P=0.0470).

It has been shown that *m-numb*, *p21* and *c-mos* were the Msi1 target genes in which there is a binding sequence region (G/A)U_1-3_AGU in their 3′-terminal untranslated region [28, 30, 34]. Using DNAMAN software, the Msi1 binding site and its hairpin structure in p21 protein are shown in Fig. 6A; the 3′UTR mRNAs of the p27 and p53 genes contain two Msi1 binding sequences (p27–1, p27–2, p53–1 and p53–2), and their secondary structures are also shown in Fig. 6A, respectively. To test if these predicted Msi1-binding sites of p21, p27, and p53 could be occupied and their translation could been inhibited by Msi1 in cervical cancer cells, the wild-type 3′UTRs of p21, p27 and p53 as well as their mutants were inserted downstream of Luciferase vectors separately (Fig. 6B). The dual-luciferase reporter assays with Msi1 over-expression and control HeLa and SiHa cells are shown in Fig. 6C and 6D, respectively. After transfection by the p21 wild-type vector, the luciferase activity in Msi1 over-expressing SiHa and HeLa cells were significantly lower than in the control cells (P<0.05). However, after being transfected with the p21 mutant vector, the difference in the luciferase activities between the Msi1 over-expressing SiHa and HeLa cells and their control cells was diminished. These results suggested that Msi1 might inhibit p21 translation by occupying a specific binding sequence in cervical cancer cells. A similar result was reported by Martin Gotto in endometrial carcinoma cells[28]. Furthermore, the luciferase activity in Msi1 over-expressing SiHa and HeLa cells was much lower than that in their control cells when transfected with both of the p27 wild-type vectors (P<0.05). However, after being transfected with the p27–1 and p27–2 mutant vectors, the luciferase activities lost significant differences between the Msi1 over-expressing SiHa and HeLa cells and their control cells, suggesting that Msi1 might repress p27 translation by occupying the p27–1 and/or p27–2 sequences in cervical cancer cells. Similarly, the luciferase activity levels in Msi1 over-expressing SiHa and HeLa cells were found to be lower than their control cells after being transfected with the p53 wild-type vector (P<0.05); however, the differences were not significant between the Msi1 over-expressing SiHa and HeLa cells and their control cells after being transfected by the p53–1 mutant vector. The differences persisted when the cells were transfected by p53–2 and p53 1&2 mutant vectors, suggesting that Msi1 might inhibit p53 translation by specifically binding to p53–1, although not to p53–2, in cervical cancer cells. Furthermore, results from RNA-protein pull-down assay (Fig.6E) showed that Msi1 protein could be strongly associated at all 3′UTR binding sites of p21, p27 and p53 (p21, p27–1, p27–2, p53–1 and p53–2) in HeLa-Msi1 cells. However, in SiHa-Msi1 cells, Msi1 protein kept a strong binding at 3′UTR binding sites of p21, p27–1 and p53–1; but kept a relatively weak binding at the sites of p27–2 and p53–2, suggesting that Msi1 may have the different effects on target genes in different cervical cancer cell lines. All these results indicated that Msi1 could bind to the binding sites of p21, p27 as well as p53.

To support the hypothesis that Msi1 could possibly down-regulate the expression of p21, p27 and p53 in cervical carcinoma tissues, the expression levels of Msi1 and p21, p27, p53 in 13 human cervical cancer samples were examined using immunohistochemistry in 4-μM consecutive tissue sections (Fig. 6F). The correlation tests demonstrated that Msi1 maintained a negative correlation with p21 expression (Fig. 6G; r=-0.5907, P=0.0335), with p27 (Fig. 6H; r=-0.5586, P=0.0472), and with p53 (Fig. 6I; r=-0.5590, P=0.0470). These results indirectly suggested that Msi1 might be a negative regulator of p21, p27 and p53 in cervical cancer tissues.

**Figure 7:**
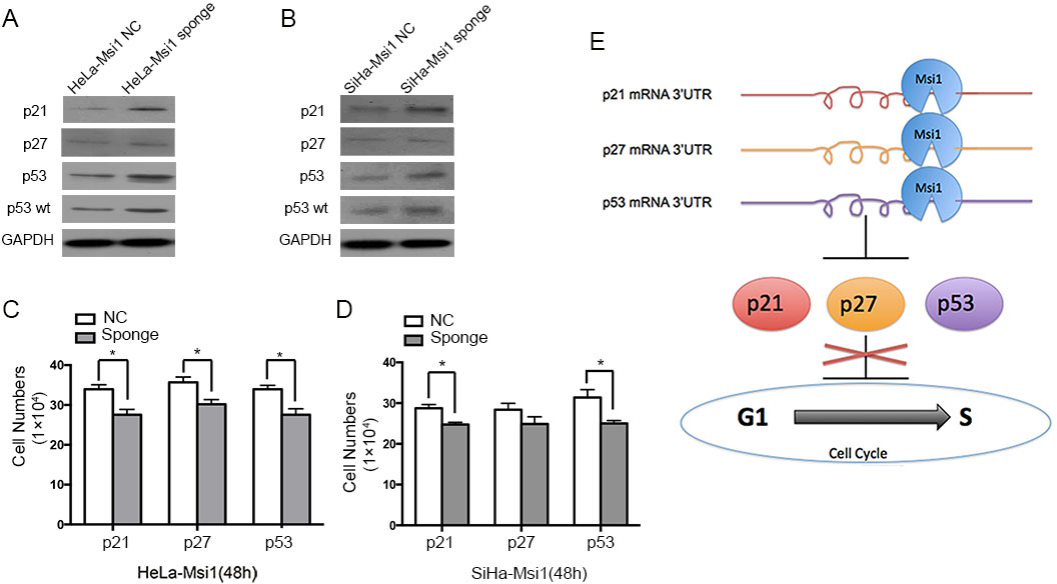
The expression of p21, p27 and p53 could be rescued by overexpressing the related Msi1 sponge vectors, respectively Western blot experiment shows the expression of p21, p27, p53 and wild type p53 (p53 wt) proteins after transfecting the corresponding Msi1 sponge vectors for 48 hours containing 2×binding sites fragments of p21, p27 and p53 in Msi1 overexpressing HeLa **(A)** and SiHa cells **(B)**. Cell proliferations after transfecting the corresponding Msi1 sponge vectors for 48 hours was determined by counting the cells for HeLa-Msi1 cells **(C)** and SiHa-Msi1 cells **(D)**. The data are shown as the mean± S.E.M. * P < 0.05. **(E)** The summary for the mechanism that Msi1 accelerates cell proliferation and enhances the progression of cervical carcinoma by directly down-regulating the translation of p21, p27 and p53.

At last, in order to identify the effects on the proliferation of cervical carcinoma cells of the functional p21, p27 and p53 exerted by Msi1, rescue treatments were performed by transfecting Msi1 sponge vectors containing 2×binding sites fragments of p21, p27 and p53 in both Msi1 overexpressing HeLa (Fig.7A) and SiHa cells (Fig.7B). The expression of p21 and p53 proteins were significantly increased, but p27 protein expression was weakly increased, after transfected the corresponding Msi1 sponge vectors for 48 hours in both HeLa-Msi1 and SiHa-Msi1 cells. Cell proliferation assay indicated that HeLa-Msi1 cell proliferations were significantly slowed down after the transfected with Msi1 sponge vectors of p21, p27 and p53 (P<0.05;Fig.7C). The proliferation of the SiHa-Msi1 cells transfected with Msi1 sponge vectors of p21 and p53 were also significantly retarded (P<0.05;Fig.7D). After transfected with Msi1 sponge vectors of p27, the proliferation of the SiHa-Msi1 cells was decreased without statistical significance (P>0.05, Fig.7D), suggesting that Msi1 have a limited effect through p27 expression on the SiHa cells. All these data supported that Msi1 promoted the proliferation of cervical cancer cells through inhibiting the translation of p21, p53, as well as p27 proteins.

## DISCUSSION

Msi1 was first identified in *Drosophila* as a determinant of sensory organ development[35, 36]. Later, Msi1 was found to be highly expressed in neural precursor cells, including neural stem cells, and was shown to be a neural stem cell marker in mammals[37–41]. Msi1 is also thought to be a potential marker of stem cells in the colon[42]. However, recent studies determined that Msi1 plays a more important role in regulating target genes expansively and directly compared with its relation to stemness, which is involved in several posttranscriptional stages of gene expression[43]. Msi1 appears to be strongly associated with tumor progression in human somatic tumors, including endometrial cancer [27], gastric cancer[44] and esophageal adenocarcinoma[45]. In our study, Msi1 was found to be up-regulated from normal cervix, cervical carcinoma in situ, to invasive cervical carcinoma and different cervical cancer cell lines, but there no different Msi1 expression levels according to clinical pathological parameters such as age and pathological stage (supplement Fig.F). Feng et al determined that Msi1 is highly expressed in malignant cervical epithelial cells with no differences between Msi1 expression levels and different clinical pathological parameters[46]. All of these data indicate that Msi1 is highly expressed in human carcinogenesis and might function as a promoter in tumor development and progression.

To further explore the function of Msi1 in cervical carcinoma, exogenous Msi1 and Msi1 shRNA were separately stably transfected into two cervical cancer cell lines, HeLa and SiHa. The exogenous Msi1 overexpression in HeLa and SiHa cells enhanced tumor formation in vivo and cell proliferation *in vitro* as well as in vivo. Furthermore, the down-regulation of Msi1 in HeLa and SiHa cells by shRNA inhibited the tumor formation in vivo and cell proliferation *in vitro* as well as *in vivo*. Therefore, our *in vitro* and *in vivo* experiments support the fact that Msi1 is a tumor enhancer for cervical carcinoma. Several studies have shown that Msi1 also promotes cell growth and tumor formation in colon cancer[20, 22] and lung cancer [25]. All of these data are in accordance with each other and support the idea that Msi1 is a tumor promoter in human cancers.

The cell cycle analysis showed that Msi1 over-expression accelerated the transition of cervical cancer cells from the G0/G1 phase to the S phase, whereas Msi1 down-regulation blocked the transition of cervical cancer cells from the G0/G1 phase to the S phase. These results suggested that Msi1 might regulate the cell cycle through cell cycle checkpoint proteins because Msi1 could bind to mRNA to interfere with protein translation. In our tested cell cycle checkpoint proteins, the expression of p21, p27 and p53 were down-regulated in Msi1 over-expressing cervical cancer cells and up-regulated in the shRNA transfected cervical cancer cells, suggesting that the cell cycle transition from the G0/G1 phase to the S phase in cervical cancer cells might be regulated by Msi1 through p21, p27 and p53. p21 is a proven Msi1 target in the neural system and in some human tumors[28, 34]. Although Arbeu et al [30] used Pathway Studio software to predict that TP53 and CDKN1B (p27) might highly associate with Msi1 identified targets, there was previously no experimental evidence to support this prediction. Therefore, this is the first study to support the idea that p53 and p27 are targets of Msi1.

To further confirm that Msi1 could regulate p21, p27 and p53 by binding to the mRNA of these genes in cervical cancer cells, pMIR-REPORT luciferase vectors containing the 3′UTRs of the p21, p27 and p53 proteins were transfected to Msi1 overexpressing cells. A dual-luciferase report assay revealed that, in cervical cancer cells, Msi1 could bind to the 3′UTRs of p21, p27 and p53 and directly inhibit the translation of these proteins. However, p21 was shown to have one effective Msi1 binding site; the translation of p27 was inhibited when Msi1 binds simultaneously to both of the Msi1 binding sites of p27. One of the two Msi1 binding sites in p53 mRNA was effective for p53 translation inhibition. For further demonstration, Msi1 were pulled down by the RNAs which contained p21, p27–1, p27–2, p53–1 and p53–2 binding sequence by RNA-protein pull-down assays in cervical cancer cells, indicating that Msi1 could bind to all the binding sites in different degrees. The expression of the Msi1, p21, p27 and p53 proteins by immunohistochemistry showed that Msi1 was negatively related to p21, p27 and p53, indirectly suggesting that Msi1 negatively regulates p21, p27 and p53 in human cervical cancer samples. These results suggest that Msi1 accelerates cell proliferation and enhances the progression of cervical carcinoma by directly down-regulating the translation of p21, p27 and p53 (Fig. 7E). Msi1 might be an important protein in controlling the progression of cervical carcinoma. Finally, results of rescue treatments by transfecting Msi1 sponge vectors to cervical cancer cells revealed that the expression of p21, p27 and p53 were rescued by overespressing the related sponge vectors, respectively. It was reported that Msi1 modulated cell cycle progression and apoptosis via Notch-1 and p21 in endometrial carcinoma [30], and Msi1 modulated mammary progenitor cell expansion through the activation of the Wnt and Notch pathways [17]. Msi1 expression was also proved to be enhanced when Hedgehog signals were activated [16]. As far as we know, this is the first paper to demonstrate that Msi1 is a tumor enhancer in cervical carcinogenesis. For the first time, p27 and p53 were identified to be targets of the Msi1 protein. Our study suggested that Msi1 might be an target in the molecular therapy for cervical carcinoma.

## MATERIALS AND METHODS

### Tissue specimens and cell lines

All study procedures followed medical ethics approval practices. One hundred and nineteen clinical specimens, comprising 30 samples of NC epithelia, 30 samples of CIS and 59 samples of ICC, were collected from patients at the First Affiliated Hospital of Xi'an Jiaotong University Medical College from 2005 to 2013. None of the patients had received chemotherapy, immunotherapy or radiotherapy before the specimen collection. The histological classifications and clinical staging were based on the International Federation of Gynecology and Obstetrics classification system.

The human cervical cancer cell lines HeLa, SiHa, C-33 A, and Caski and the human teratoma cell line PA-1 were cultured in our lab. Dulbecco's modified Eagle's medium (DMEM; Sigma-Aldrich, USA) was used to culture HeLa, SiHa, C-33 A and PA-1 cells at 37°C with 5% CO2. RPMI 1640 (Sigma-Aldrich, St. Louis, MO) were used for the Caski cells under the identical conditions. The media were supplemented with 10% fetal bovine serum (FBS; Invitrogen, Carlsbad, CA).

### Western blotting

The cell lysates were produced in ice using a lysis buffer (150 mM NaCl; 50 mM Tris-HCl, pH 7.4; 2 mM EDTA; 1% NP-40; and 0.1% SDS) containing a protease inhibitor cocktail (Complete Mini; Roche Diagnostics, Branchburg, NJ, USA). The protein concentrations were determined using the BCA Protein Assay Reagent (Pierce, Rockford, IL, USA). The protein samples (20 μg) were separated using SDS-PAGE and transferred to polyvinylidenedifluoride membranes (Millipore, Billerica, MA, USA). The appropriate primary antibodies were used after the membranes were blocked in 5% fat-free milk. The primary antibodies were anti-Msi1 (1:1000, #H00004440-M04, Abnova), anti-CDK2 (1:500 dilution, sc-163, Santa Cruz Biotechnology), anti-cyclinA (1:500, sc-596, Santa Cruz Biotechnology), anti-cyclinD1 (1:500 dilution, sc-8396, Santa Cruz Biotechnology), anti-p21 (1:500, sc-6246, Santa Cruz Biotechnology), anti-p27 (1:500, sc-528, Santa Cruz Biotechnology), anti-p53 (1:500, sc-6246, Santa Cruz Biotechnology), anti-p53(wild type)(1:500, MABE339, Millipore) and anti-β-actin (1:1000, sc-47778, Santa Cruz Biotechnology). The proteins were detected by ECL detection (Millipore) after using a secondary antibody coupled with horseradish peroxidase (Thermo Fisher Scientific Inc., New York, NY, USA) and visualized on X-ray film. The relative densities of the Western blot bands were measured using the Alpha View system (Cell Biosciences, Santa Clara, CA, USA).

### Immunostaining

Formalin-fixed, paraffin-embedded, 4-μm sections of tissue samples were deparaffinized and rehydrated and then treated with citrate buffer (10 mM sodium citrate, 2 mM citric acid, pH 6.0) in a pressure cooker for 2 min, and then with 3% H_2_O_2_ at room temperature for 10 min. After washing with phosphate-buffered saline (PBS) at room temperature, the sections were incubated with the following primary antibodies at 4°C overnight: anti-Msi1 (1:100, #H00004440-M04, Abnova); anti-p21 (1:50, sc-6246, Santa Cruz Biotechnology); anti-p27 (1:50, sc-528, Santa Cruz Biotechnology); anti-p53 (1:50, sc-6246, Santa Cruz Biotechnology). The sections were then incubated with a horseradish peroxidase-conjugated secondary antibody and visualized with the treatment of 3,3′-diaminobenzidine under the microscope. The nuclei were counterstained with hematoxylin. Negative controls were performed in the same manner, except that PBS was used as a substitute for the primary antibody. The IHC was examined by two separate researchers using an Olympus-CX31 microscope(Olympus, Tokyo, Japan) in five randomly selected representative fields at ×40 magnification. The evaluation of Msi1, p21, p27 and p53 staining were performed using the immunoreactivity scores(IRS). The score was determined by multiplying the staining intensity by the staining extent. The staining intensity for Msi-1 was scored as 0 (negative), 1 (weak), 2 (moderate), and 3 (strong). The staining extent was scored as 0 (0%), 1 (1%–25%), 2 (26%–50%), 3 (51%–75%), and 4 (76%–100%) according to the percentage of positively stained cells[20]. The Msi1 staining was defined into two categories according to IRS: negative (1–4) and positive (5–12).

For the immunocytochemistry experiments, the cells were cultured on autoclaved cover slips, fixed in 4% paraformaldehyde, treated with 0.2% Triton X-100 for 20 min at room temperature, and then incubated with the primary antibodies described above.

### PCR analysis

The total RNA was extracted from cells using the TRIzol reagent (Invitrogen). Cell cycle-related genes were first subjected to reverse transcription-PCR (RT-PCR) using an RT-PCR kit (MBI, Hanover, MD, USA) and then subjected to quantitative real-time RT-PCR using the iQ5 Real-Time PCR Detection System (Bio-Rad, Hercules, CA, USA) with SYBR Premix Ex Taq II (TaKaRa). GAPDH mRNA levels were used as an internal normalization control. Fold changes were calculated and normalized using the CT method. PCR was performed with the Taq PCR Master Mix (Tian Gen, Beijing, China).

### Plasmid construction

The Msi1 CDS was amplified from SiHa cDNA using the primers listed below and cloned into the pCAG-IRES2-AcGFP1 expression vector, which was generated by replacing the CMV promoter of pIRES2-AcGFP1 (Clontech, Mountain View, CA, USA) with the CAG promoter of the pCAG-GFP vector (Addgene, Cambridge, MA, USA).

To construct a three reporter vector containing the wild-type 3′UTR of p21, p27 and p53, a 1.5 kb fragment of the 3′UTR of p21 mRNA, a 1.2 kb fragment of the 3′UTR of p27 mRNA and a 1 kb fragment of the 3′UTR of p53 mRNA were separately extracted from HeLa cells using the primers listed below and amplified by PCR and then cloned into the pMIR-REPORT luciferase vector downstream of the Luciferase gene.

In the mutation analysis, the binding sequence of the p21 3′UTR (GTAGT) was replaced by the random bases (AGCAG), the p27 3′UTR (ATAGT) and (GTAGT) were replaced by the random bases (CGCAG) and (AGCAG), respectively, the p53 3′UTR (GTTAGT) and (GTAGT) were replaced by the random bases (AGGCAG) and (AGCAG), respectively.

To construct Msi1 sponge vectors containing p21, p27 and p53 binding sites, two equal 250bp fragments of the 3′UTR of p21 mRNA with different restriction sites, two 400bp fragments of 3′UTR of p27 mRNA with different restriction sites and two 400bp fragments of 3′UTR of p53 mRNA with different restriction sites were separately extracted from HeLa cells using the primers listed below and amplified by PCR and then cloned into the IRES-AcGFP vectors, respectively.

Msi1 F: 5′-CCTAAGCTTCCGATGGAGACTGACGCGC-3′;

Msi1 R: 5′-TCCGGATCCTCAGTGGTACCCATTGGTG-3′;

p21 3′UTR F: 5′-ATTGAGCTCCGCCCACAGGAAGCCTG-3′;

p21 3′UTR R: 5′-CCGACGCGTACAAGTAAAGTCACTAAG-3′

p27 3′UTR F: 5′-CGCGAGCTCGAATTAAGAATATGTTTC-3′

p27 3′UTR R: 5′-TTGACGCGTATGCAACCTTTTAAGCATAGC-3′

p53 3′UTR F: 5′-CCTGAGCTCCACTTCTTGTTCCCCACTG-3′

p53 3′UTR R: 5′-TTGACGCGTGGTGGCTCACAATTGTAATC-3′

p21 fragment1 F: 5′-CGAGCTCAGCGACCTTCCTCATCCAC-3′

p21 fragment1 R: 5′-CGGAATTCCAGTCCAGGCCAGTATGTTAC-3′

p21 fragment2 F: 5′-CGGAATTCAGCGACCTTCCTCATCCAC-3′

p21 fragment2 R: 5′-CGGGATCCCAGTCCAGGCCAGTATGTTAC-3′

p27-1&2 fragment1 F: 5′-CCGCTCGAGTGATCTGCCTCTAAAAGCGT-3′

p27-1&2 fragment1 R: 5′-CGGAATTCATTCTTAACATTCAAAACTCCC-3′

p27-1&2 fragment2 F: 5′-CGGAATTCTGATCTGCCTCTAAAAGCGT-3′

p27-1&2 fragment2 R: 5′-CGGGATCCATTCTTAACATTCAAAACTCCC-3′

p53-1&2 fragment1 F: 5′-CCGCTCGAGGGCTGGGCCAGCAGAGACTTGAC-3′

p53-1&2 fragment1 R: 5′-CGGAATTCAGGAGGATGGGGAGTAGGACATACC -3′

p53-1&2 fragment2 F: 5′-CGGAATTCGGCTGGGCCAGCAGAGACTTGAC-3′

p53-1&2 fragment2 R: 5′-CGGGATCCAGGAGGATGGGGAGTAGGACATACC -3′

### Cell growth and cell viability assays

Cells (5×10^4^) were seeded in triplicate with 2 mL of media into 35-mm tissue culture dishes for 7 days. The numbers of cells were counted after harvesting every two days using a hemocytometer under light microscopy.

The cell viability assays were performed by applying 3-(4,5-dimethylthiazole-yl)-2,5-diphenyl tetrazolium bromide (Sigma-Aldrich) dye to cells that were seeded in 96-well plates for 1000 cells each, as described in a standard protocol. Then, the absorbance at 490 nm was measured (Bio-Rad).

### Flow cytometry analysis

Cells were harvested and fixed with 70% cold ethanol at 4°C overnight. After being washed in PBS, the cells were incubated in 1 mL of staining solution (20 mg/mL propidium iodide; 10 U/mL RNaseA) at room temperature for 30 min. Then, the samples were measured by FACS Calibur flow cytometry (Becton Dickinson, Franklin Lakes, NJ, USA), and the cell cycle distributions were analyzed by the software ”Flowjo’’ (Verity Software House).

### Tumor xenograft assays

The experimental protocols used were evaluated and approved by the Animal Care and Use Committee of the Medical School of Xi'an Jiaotong University. Groups of 4- to 6-week-old Balb/c athymic nude mice were subjected to infections in subcutaneous sites with 1×10^6^ HeLa cells or 1×10^6^ SiHa cells and housed in a pathogen-free facility. The tumor dimensions were measured every three days, and the volumes were calculated by the standard formula: length×width^2^/2. At the end of the experiment, the mice were killed by cervical vertebra dislocation, and the weights of the tumors were measured after being dissected out.

### RNA-protein pull-down assay

The assay was conducted with a RNA-Protein Pull-Down Kit (Pierce, Rockford, IL, USA). 50bp single strand RNAs with the relative binding sequences of p21, p27–1, p27–2, p53–1 and p53–2 were synthesized at first. The supplied RNAs (50pmol) were labeled using desthiobiotinylated cytidine bisphosphate and T4 RNA ligase. Labeled RNA was captured using 50μL of streptavidin magnetic beads in RNA Capture Buffer for 30 minutes at room temperature. Beads were washed twice in 20mM Tris (pH 7.5), once in Protein-RNA Binding Buffer and 100μg of protein extract was added. Samples were incubated for 45 minutes at 4°C, washed three times with Wash Buffer and eluted after 15 minutes of incubation at 37°C with Biotin Elution Buffer. RNA pull-down specificity was assessed by Western blotting with samples normalized by volume and bands detected.

### Luciferase reporter assay

In brief, plasmids containing firefly luciferase reporters were co-transfected into cells using Lipofectamine 2000 (Invitrogen). After being incubated for 48 h, the cell monolayers were harvested by resuspension in passive lysis buffer. Luciferase activity was determined in a luminometer (Promega). The efficiency of transfection was normalized to the paired Renilla luciferase activity using the Dual Luciferase Reporter Assay System (Promega). The specific activity was displayed as the fold change of the experimental group versus the control group.

### Statistical analysis

Student's t-test or a one-way ANOVA test were used for univariate analyses. A two-way ANOVA test was used to determine the differences in the volume in the tumor xenograft assay between the groups. Pearson's correlation test was used to consider correlations between Msi1 and p21, p27 and p53. Statistical analyses were performed using the Statistical Package of Social Science (SPSS) software, version 18.0 (SPSS Inc., Chicago, IL, USA). All of the tests were two-sided. A P value <0.05 was considered statistically significant.

## SUPPLEMENTARY FIGURE



## References

[1] Jemal A, Bray F, Center MM, Ferlay J, Ward E, Forman D (2011). Global cancer statistics. CA Cancer J Clin.

[2] Walboomers JMM, Jacobs MV, Manos MM, Bosch FX, Kummer JA, Shah KV, Snijders PJF, Peto J, Meijer CJLM, Munoz N (1999). Human papillomavirus is a necessary cause of invasive cervical cancer worldwide. Journal of Pathology.

[3] Liu XF, Yang WT, Xu R, Liu JT, Zheng PS (2014). Cervical Cancer Cells with Positive Sox2 Expression Exhibit the Properties of Cancer Stem Cells. Plos One.

[4] Liu SY, Zheng PS (2013). High aldehyde dehydrogenase activity identifies cancer stem cells in human cervical cancer. Oncotarget.

[5] Lopez J, Poitevin A, Mendoza-Martinez V, Perez-Plasencia C, Garcia-Carranca A (2012). Cancer-initiating cells derived from established cervical cell lines exhibit stem-cell markers and increased radioresistance. BMC Cancer.

[6] Wu XL, Zheng PS (2013). Undifferentiated embryonic cell transcription factor-1 (UTF1) inhibits the growth of cervical cancer cells by transactivating p27Kip1. Carcinogenesis.

[7] Wang YD, Cai N, Wu XL, Cao HZ, Xie LL, Zheng PS (2013). OCT4 promotes tumorigenesis and inhibits apoptosis of cervical cancer cells by miR-125b/BAK1 pathway. Cell Death & Disease.

[8] Ji J, Zheng PS (2010). Expression of Sox2 in human cervical carcinogenesis. Human Pathology.

[9] Gu TT, Liu SY, Zheng PS (2012). Cytoplasmic NANOG-positive stromal cells promote human cervical cancer progression. Am J Pathol.

[10] Yang WT, Zheng PS (2012). Kruppel-like factor 4 functions as a tumor suppressor in cervical carcinoma. Cancer.

[11] Imai T, Tokunaga A, Yoshida T, Hashimoto M, Mikoshiba K, Weinmaster G, Nakafuku M, Okano H (2001). The neural RNA-binding protein Musashi1 translationally regulates mammalian numb gene expression by interacting with its mRNA. Molecular and Cellular Biology.

[12] Good P, Yoda A, Sakakibara S, Yamamoto A, Imai T, Sawa H, Ikeuchi T, Tsuji S, Satoh H, Okano H (1998). The human Musashi homolog 1 (MSI1) gene encoding the homologue of Musashi/Nrp-1, a neural RNA-binding protein putatively expressed in CNS stem cells and neural progenitor cells. Genomics.

[13] Toda M, Iizuka Y, Yu W, Imai T, Ikeda E, Yoshida K, Kawase T, Kawakami Y, Okano H, Uyemura K (2001). Expression of the neural RNA-binding protein Musashi1 in human gliomas. Glia.

[14] Dahlrot RH, Hansen S, Herrstedt J, Schroder HD, Hjelmborg J, Kristensen BW (2013). Prognostic value of Musashi-1 in gliomas. Journal of Neuro-Oncology.

[15] Sakakibara S, Nakamura Y, Yoshida T, Shibata S, Koike M, Takano H, Ueda S, Uchiyama Y, Noda T, Okano H (2002). RNA-binding protein Musashi family: Roles for CNS stem cells and a subpopulation of ependymal cells revealed by targeted disruption and antisense ablation. Proceedings of the National Academy of Sciences of the United States of America.

[16] Hong IS, Kang KS (2013). The Effects of Hedgehog on the RNA-Binding Protein Msi1 in the Proliferation and Apoptosis of Mesenchymal Stem Cells. Plos One.

[17] Wang XY, Yin Y, Yuan H, Sakamaki T, Okano H, Glazer RI (2008). Musashi1 modulates mammary progenitor cell expansion through proliferin-mediated activation of the Wnt and Notch pathways. Mol Cell Biol.

[18] Sanchez-Diaz PC, Burton TL, Burns SC, Hung JY, Penalva LO (2008). Musashi1 modulates cell proliferation genes in the medulloblastoma cell line Daoy. BMC Cancer.

[19] Vo DT, Subramaniam D, Remke M, Burton TL, Uren PJ, Gelfond JA, de Sousa Abreu R, Burns SC, Qiao M, Suresh U, Korshunov A, Dubuc AM, Northcott PA, Smith AD, Pfister SM, Taylor MD (2012). The RNA-binding protein Musashi1 affects medulloblastoma growth via a network of cancer-related genes and is an indicator of poor prognosis. Am J Pathol.

[20] Li D, Peng X, Yan D, Tang H, Huang F, Yang Y, Peng Z (2011). Msi-1 is a predictor of survival and a novel therapeutic target in colon cancer. Ann Surg Oncol.

[21] Fan LF, Dong WG, Jiang CQ, Xia D, Liao F, Yu QF (2010). Expression of putative stem cell genes Musashi-1 and beta1-integrin in human colorectal adenomas and adenocarcinomas. Int J Colorectal Dis.

[22] Rezza A, Skah S, Roche C, Nadjar J, Samarut J, Plateroti M (2010). The overexpression of the putative gut stem cell marker Musashi-1 induces tumorigenesis through Wnt and Notch activation. Journal of Cell Science.

[23] Wang T, Ong CW, Shi J, Srivastava S, Yan B, Cheng CL, Yong WP, Chan SL, Yeoh KG, Iacopetta B, Salto-Tellez M, Consortium SGC (2011). Sequential expression of putative stem cell markers in gastric carcinogenesis. British Journal of Cancer.

[24] Nikpour P, Emadi-Baygi M, Mohhamad-Hashem F, Maracy MR, Haghjooy-Javanmard S (2013). MSI1 overexpression in diffuse type of gastric cancer. Pathol Res Pract.

[25] Wang XY, Yu H, Linnoila RI, Li L, Li D, Mo B, Okano H, Penalva LOF, Glazer RI (2013). Musashi1 as a potential therapeutic target and diagnostic marker for lung cancer. Oncotarget.

[26] Wang XY, Penalva LOF, Yuan HY, Linnoila RI, Lu JC, Okano H, Glazer RI (2010). Musashi1 regulates breast tumor cell proliferation and is a prognostic indicator of poor survival. Molecular Cancer.

[27] Gotte M, Wolf M, Staebler A, Buchweitz O, Kelsch R, Schuring AN, Kiesel L (2008). Increased expression of the adult stem cell marker Musashi-1 in endometriosis and endometrial carcinoma. Journal of Pathology.

[28] Gotte M, Greve B, Kelsch R, Muller-Uthoff H, Weiss K, Kiesel L (2011). The Adult Stem Cell Marker Musashi-1 Modulates Endometrial Carcinoma Cell Cycle Progression, Apoptosis and Tumor Growth In Vivo Via Notch-1 and p21WAF1/CIP1. Reproductive Sciences.

[29] Chen YZ, Wang JH, Yan J, Liang Y, Zhang XF, Zhou F (2014). Increased expression of the adult stem cell marker Musashi-1 in the ectopic endometrium of adenomyosis does not correlate with serum estradiol and progesterone levels. Eur J Obstet Gynecol Reprod Biol.

[30] Abreu RD, Sanchez-Diaz PC, Vogel C, Burns SC, Ko DJ, Burton TL, Vo DT, Chennasamudaram S, Le SY, Shapiro BA, Penalva LOF (2009). Genomic Analyses of Musashi1 Downstream Targets Show a Strong Association with Cancer-related Processes. Journal of Biological Chemistry.

[31] Sakakibara S, Imai T, Hamaguchi K, Okabe M, Aruga J, Nakajima K, Yasutomi D, Nagata T, Kurihara Y, Uesugi S, Miyata T, Ogawa M, Mikoshiba K, Okano H (1996). Mouse-Musashi-1, a neural RNA-binding protein highly enriched in the mammalian CNS stem cell. Dev Biol.

[32] Okano H, Imai T, Okabe M (2002). Musashi: a translational regulator of cell fate. Journal of Cell Science.

[33] Okano H, Kawahara H, Toriya M, Nakao K, Shibata S, Imai T (2005). Function of RNA-binding protein Musashi-1 in stem cells. Experimental Cell Research.

[34] Battelli C, Nikopoulos GN, Mitchell JG, Verdi JM (2006). The RNA-binding protein Musashi-1 regulates neural development through the translational repression of p21WAF-1. Mol Cell Neurosci.

[35] Nakamura M, Okano H, Blendy JA, Montell C (1994). Musashi, a Neural Rna-Binding Protein Required for Drosophila Adult External Sensory Organ Development. Neuron.

[36] Glazer RI, Wang XY, Yuan HY, Yin YZ (2008). Musashi1: A stem cell marker no longer in search of a function. Cell Cycle.

[37] Good P, Yoda A, Sakakibara S, Yamamoto A, Imai T, Sawa H, Ikeuchi T, Tsuji S, Satoh H, Okano H (1998). The human Musashi homolog 1 (MSI1) gene encoding the homologue of Musashi/Nrp-1, a neural RNA-binding protein putatively expressed in CNS stem cells and neural progenitor cells. Genomics.

[38] Imai T, Tokunaga A, Yoshida T, Hashimoto M, Mikoshiba K, Weinmaster G, Nakafuku M, Okano H (2001). The neural RNA-binding protein Musashi1 translationally regulates mammalian numb gene expression by interacting with its mRNA. Mol Cell Biol.

[39] Siddall NA, McLaughlin EA, Marriner NL, Hime GR (2006). The RNA-binding protein Musashi is required intrinsically to maintain stem cell identity. Proceedings of the National Academy of Sciences of the United States of America.

[40] Kawahara H, Okada Y, Imai T, Iwanami A, Mischel PS, Okano H (2011). Musashi1 Cooperates in Abnormal Cell Lineage Protein 28 (Lin28)-mediated Let-7 Family MicroRNA Biogenesis in Early Neural Differentiation. Journal of Biological Chemistry.

[41] Dobson NR, Zhou YX, Flint NC, Armstrong RC (2008). Musashi1 RNA-binding protein regulates oligodendrocyte lineage cell differentiation and survival. Glia.

[42] Akasaka Y, Saikawa Y, Fujita K, Kubota T, Ishikawa Y, Fujimoto A, Ishii T, Okano H, Kitajima M (2005). Expression of a candidate marker for progenitor cells, Musashi-1, in the proliferative regions of human antrum and its decreased expression in intestinal metaplasia. Histopathology.

[43] Galante PA, Sandhu D, de Sousa Abreu R, Gradassi M, Slager N, Vogel C, de Souza SJ, Penalva LO (2009). A comprehensive in silico expression analysis of RNA binding proteins in normal and tumor tissue: Identification of potential players in tumor formation. RNA Biol.

[44] Kuang RG, Kuang Y, Luo QF, Zhou CJ, Ji R, Wang JW (2013). Expression and significance of Musashi-1 in gastric cancer and precancerous lesions. World J Gastroenterol.

[45] Bobryshev YV, Freeman AK, Botelho NK, Tran D, Levert-Mignon AJ, Lord RV (2010). Expression of the putative stem cell marker Musashi-1 in Barrett's esophagus and esophageal adenocarcinoma. Dis Esophagus.

[46] Ye F, Zhou CY, Cheng Q, Shen JJ, Chen HZ (2008). Stem-cell-abundant proteins Nanog, Nucleostemin and Musashi1 are highly expressed in malignant cervical epithelial cells. Bmc Cancer.

