# Supplementary material for: Msi1 promotes tumor growth and cell proliferation by targeting cell cycle checkpoint proteins p21, p27 and p53 in cervical carcinomas
Source: Oncotarget. 2014 Oct 24;5(21):10870–85. doi: 10.18632/oncotarget.2539 (PMC4279416; doi:10.18632/oncotarget.2539)
Supplement: Supplementary file 1 [file oncotarget-05-10870-s001.pdf]

SUPPLEMENTARY FIGURE

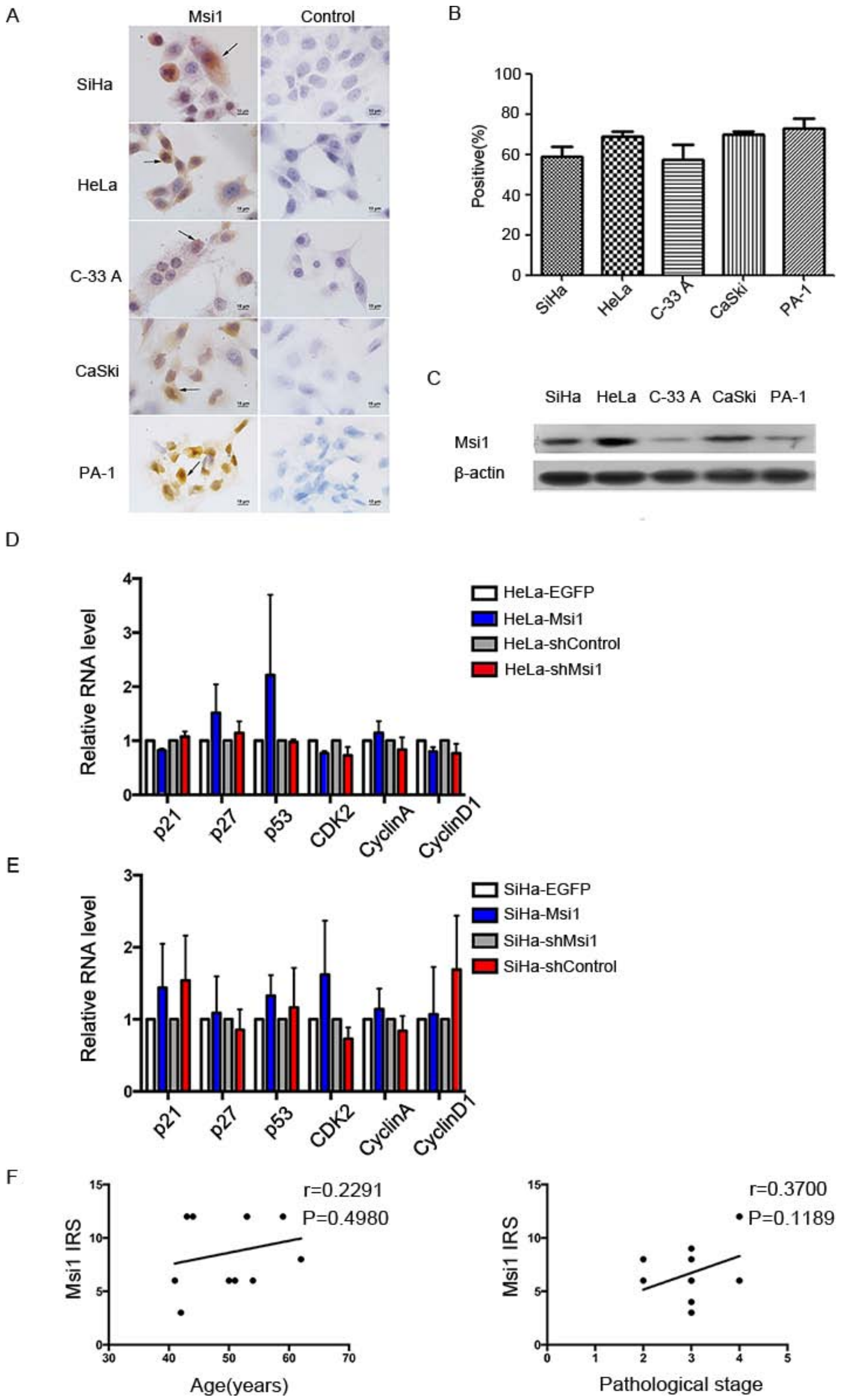

Supplementary Figure S1: The expression of Msi1 in cervical cancer cell lines, relative RNA levels of several key cell cycle regulators in Msi1-modified cervical cancer cells, as well as the difference of Msi1 level in different clinical pathological parameters such as age and pathological stage. (A) Immunocytochemistry for Msi1 protein in cervical cancer cells is shown; original magnification, ×1000. **(B)** The positive rates were summarized. The arrow indicates the Msi1 positive cell. **(C)** Msi1 expression is shown at the protein level by Western blot analysis in cervical cancer cell lines, and β-actin served as the loading control. The relative mRNA levels of several key cell cycle regulators(p21, p27, p53, CDK2, CyclinA and CyclinD1) in Msi1-modified HeLa cells **(D)** and in Msi1-modified SiHa cells **(E)** as determined by quantitative real-time–PCR. **(F)** The correlation between the age and the expression level of Msi1(left; r=0.2291, P=0.4980), pathological phases and the expression level of Msi1(right; r=0.3700, P=0.1189).
